# *Castanea sativa* Mill. By-Products: Investigation of Potential Anti-Inflammatory Effects in Human Intestinal Epithelial Cells

**DOI:** 10.3390/molecules29163951

**Published:** 2024-08-21

**Authors:** Carola Pozzoli, Giulia Martinelli, Marco Fumagalli, Chiara Di Lorenzo, Nicole Maranta, Luca Colombo, Stefano Piazza, Mario Dell’Agli, Enrico Sangiovanni

**Affiliations:** 1 Department of Pharmacological and Biomolecular Sciences “Rodolfo Paoletti” (DiSFeB), Università degli Studi di Milano, 20133 Milan, Italy; carola.pozzoli@unimi.it (C.P.); giulia.martinelli@unimi.it (G.M.); marco.fumagalli3@unimi.it (M.F.); chiara.dilorenzo@unimi.it (C.D.L.); nicole.maranta@unimi.it (N.M.); enrico.sangiovanni@unimi.it (E.S.); 2 Consorzio Castanicoltori di Brinzio, Orino e Castello Cabiaglio, Società Cooperativa Agricola-Varese, 21100 Varese, Italy; luca@artemideambiente.it

**Keywords:** *Castanea sativa* Mill., chestnut, polyphenols, inflammation, gut

## Abstract

*Castanea sativa* Mill. (*C. sativa*) processing and pruning generate several by-products, including leaves, burs, and shells (inner and outer teguments), which are considered an important source of high-value phytochemicals. Ellagitannins from *C. sativa* leaf extracts have been described to impair *H. pylori* viability and inflammation in gastric cells. Furthermore, chestnut shells showed an important anti-inflammatory effect in gastric epithelial cells. Dietary polyphenols, including tannins, have been reported to interfere with targets of inflammation, including the nuclear factor κB (NF-κB). A promising role as a further therapeutical target for gut disorders has been recently proposed for the regulatory subunit of hypoxia-inducible factor (HIF-1α), as a potential stabilizer of intestinal barrier integrity. Therefore, the main objective of this work is the chemical characterization of several chestnut by-products (bud, spiny bur, wood, pericarp and episperm), together with the exploitation of their anti-inflammatory properties in intestinal cells, scavenging capacity, and stability following gastrointestinal digestion. The chemical characterization confirmed the presence of bioactive polyphenols in the extracts, including ellagitannins. In CaCo-2 cells stimulated by an IL-1β-IFN-γ cocktail, nearly all chestnut by-products (50 µg/mL) inhibited the release of proinflammatory mediators (CXCL-10, IL-8, MCP-1, ICAM), along with the NF-κB-driven transcription, and induced the HRE-driven transcription. The stability of the most promising extracts, identified through PCA and cluster analysis, was addressed by in vitro gastrointestinal digestion. Despite the significant reduction in total polyphenol index of chestnut bud and wood after gastric and intestinal digestion, the activity of these extracts on both scavenging and anti-inflammatory parameters remained promising. These data contribute to exploit the potential of chestnut by-products as sources of dietary polyphenols with anti-inflammatory properties at the intestinal level. Moreover, this study could represent an important step to encourage the recycling and valorization of chestnut by-products, promoting the circular economy and reducing the environmental impact related to the management of agriculture waste.

## 1. Introduction

The genus *Castanea*, a member of the Fagaceae family, is native to South Europe and Asia. Recently, there has been a growing interest in the cultivation of European chestnut (*Castanea sativa* Mill.) in the Mediterranean region, driven by its inclusion in the human diet and the occurrence of several phytochemicals with a wide range of biological effects. According to data from the Food and Agriculture Organization of the United Nations (FAO), the harvest of sweet chestnuts nearly doubled from 2016 to 2019, reaching approximately 303,100 tons in the Mediterranean area. During this period, Spain, Italy, and Portugal emerged as the leading producers [1].

Throughout the entire process of fruit cultivation, harvesting, industrial processing, and consumption, substantial amounts of by-products with an important high environmental impact are generated, such as leaves, burs, and shells (inner and outer teguments). These by-products can serve as a valuable biomass for the recovery of high-value phytochemicals, thereby contributing to environmental sustainability [2,3,4].

Chestnut by-products are notable as an interesting source of polyphenols, especially tannins. Vescalagin and castalagin, which are present along with their precursors ellagic and gallic acids, represent the main hydrolysable tannins, whereas epigallocatechin, catechin, and epicatechin are the primary components of condensed tannins in chestnut by-products [3,5,6,7].

Several biological activities have been ascribed to chestnut by-products and their bioactive compounds, especially in the context of inflammation and oxidative stress. In a model of neuroinflammation, chestnut leaf and spiny bur extracts exerted anti-inflammatory activity in BV-2 microglial cells [8,9]. In another study, methanolic extracts of chestnut burs, leaves, and fruits reduced NF-κB activity in THP-1 monocytes following stimulation with LPS and showed antioxidant activity in a variety of spectrophotometric assays [10]. In other studies from our group, ellagitannins from *C. sativa* leaf extracts impaired *H. pylori* viability and inflammation in gastric epithelial cells [11]; moreover, the external parts of the chestnut fruit (pericarp and episperm) significantly reduced inflammation induced by TNFα in gastric epithelial cells [12].

In the context of inflammatory processes, several studies explored the role of polyphenol compounds, particularly those belonging to the tannin class, in both in vivo and in vitro models of intestinal diseases [13,14,15,16,17].

Inflammatory bowel diseases (IBD) are closely associated with inflammation and overproduction of reactive oxygen species (ROS), conditions often linked to the activation of nuclear factors, such as nuclear factor-κB (NF-κB), leading to increased transcription of pro-inflammatory mediators. A recent review highlighted how several phytochemicals can interfere with many IBD inflammatory targets, including the NF-κB pathway, thus reducing the transcription of pro-inflammatory mediators, such as interleukin (IL)-1β, tumor necrosis factor-α (TNF-α), and interferon (IFN)-γ [18].

Besides inflammation, new emerging redox-targeted therapeutics to counteract this disease include the family of hypoxia-inducible factors (HIFs), which act as transcription factors mediating the hypoxic response in cells and tissues. HIFs coordinate a transcriptional program involving a wide range of physiological functions, including angiogenesis, erythropoiesis, cellular metabolism, autophagy, apoptosis, and other physiological responses [19]. Different isoforms of HIF play a complex role in colitis models, which is currently under investigation [20], but activation of HIF-1α, the regulatory subunit of the transcription factor HIF-1, seems to emerge as stabilizer by improving intestinal barrier integrity during mucosal insult [21]. To the best of our knowledge, only few studies have investigated the role of natural compounds, such as polyphenols, to induce HIF-1α and regulate HIF-1 transcriptional activity [22].

Therefore, the aim of this work was to elucidate the potential anti-inflammatory properties and scavenging capacity of *C. sativa* by-products in an in vitro model of intestinal inflammation, along with the analysis of their chemical composition. Principal component analysis (PCA) was employed to identify the most promising chestnut sources following biological investigations.

## 2. Results and Discussion

### 2.1. Chemical Characterization and Scavenging Capacity

Chestnut by-products represent a valuable source of bioactive compounds, particularly polyphenols [23,24].

The total polyphenol index (TPI) in chestnut by-products was determined, and the results are reported in Table 1. Statistically significant (*p* < 0.001) differences were observed among the hydroalcoholic extracts, with the TPI ranging from 54.91 ± 4.63 (mean ± SEM) mg gallic acid equivalent/g in fresh pericarp to 428.40 ± 3.31 (mean ± SEM) mg gallic acid equivalent/g in quiescent bud.

Vella et al. [23] obtained a similar TPI for pericarp (212.82–337.33 mg GAE/g DW among several chestnut varieties) but observed lower values for bur (60.01–100.58 mg GAE/g DW) and episperm (3.62–5.95 mg GAE/g DW) extracts. These results were in line with those observed for chestnut by-products reported by Pinto et al. [25] (315.21–496.80 mg GAE/g DW for shells obtained through subcritical water extraction [SWE] under different settings), Comandini et al. [26] (239–561 mg GAE/g DW for methanolic bark) and Pinto et al. [27] (132.66 mg GAE/g DW for shells). Cerulli et al. observed even higher TPI values in methanolic extracts of burs and shells (870.81 ± 1.49 mg GAE/g for shells; 580.44 mg GAE/g for burs) [10,28].

Vescalagin and castalagin were previously identified in other works as noteworthy ellagitannins with proven anti-inflammatory activity in gastric cells [11]. Thus, the amount of these compounds in hydroalcoholic chestnut by-products was estimated by HPLC coupled with triple quadrupole MS/MS using external calibration. The results, reported in Table 1, showed that in extracts from pruning quiescent buds and fresh wood, the vescalagin content was significantly higher than in other by-products, reaching 19.21 ± 0.1 µg/g (mean ± SEM) in quiescent buds and 21.43 ± 0.1 µg/g (mean ± SEM) in fresh wood. Significant differences in castalagin content were also noted, with the highest concentrations found in fresh/dry wood and spiny burs, measuring 41.35 ± 0.2 µg/g (mean ± SEM), 39.43 ± 0.1 µg/g (mean ± SEM), and 32.38 µg/g (mean ± SEM), respectively.

Scavenging capacity is closely related to polyphenol content, with several studies suggesting a potential mechanism related to the presence and amount of hydroxyl groups in their chemical structure [29,30]. The scavenging activity of the extracts was measured and is reported in Table 1.

In line with the higher total polyphenol index and ellagitannin content observed, extracts from winter pruning exhibited higher antioxidant activity compared to those from fruit-derived by-products. The scavenging capacity in the pruning extracts (buds and wood) ranged from 46.02 to 50.97 mmol Trolox equivalent/g (ORAC method) and from 6.58. to 7.27 mmol Trolox equivalent/g (DPPH method). Total polyphenol index significantly correlated with scavenging capacity, evaluated both with DPPH (r = 0.923, *p* < 0.001) and ORAC (r = 0.768, *p* < 0.001). The results align with other studies on chestnut by-product extracts [31,32].

HPLC/LTQ ion trap/MS/MS analysis of *C. sativa* by-products enabled the putative identification of various classes of metabolites (Table 2), primarily hydrolysable tannins (derived from galloylglucose and ellagitannins) and condensed tannins (proanthocyanidins). Additionally, a small number of other molecules such as flavonoids, ellagic acid, and phenolic derivatives were identified. These chemical fingerprints align with the composition of *C. sativa* by-products reported in the literature [10,33].

### 2.2. Anti-Inflammatory Properties

To explore the potential anti-inflammatory effects of chestnut by-product extracts in an intestinal inflammation model, a pro-inflammatory environment was simulated in vitro using a combination of IL-1β and IFN-γ, both at 10 ng/mL.

IFN-γ is known to enhance the inflammatory response to innate cytokines in epithelial cells, including CaCo-2 cells [34]. According to several studies, CaCo-2 cells are mainly responsive to IL-1β [34,35]. Thus, a cocktail of IFN-γ and IL-1β was used to stimulate the release of several pro-inflammatory markers, following experimental settings previously published by our group [36].

**Figure A1:**
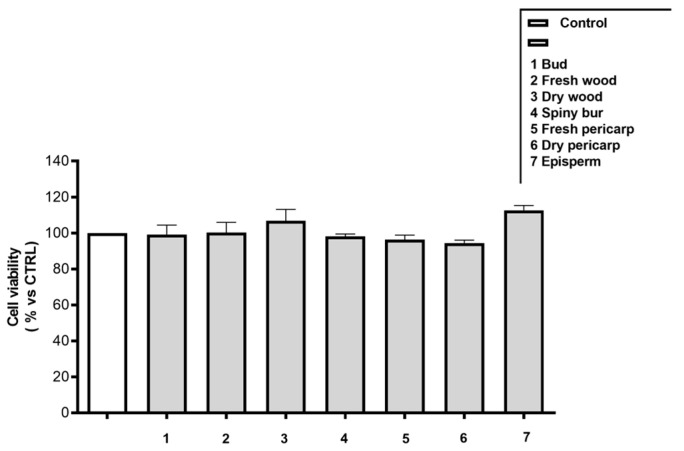
MTT assay performed on *C. sativa* by-product extracts (50 µg/mL) in CaCo-2 cells. Data (*n* = 3) are expressed as mean (%) ± SEM relative to control, which was arbitrarily assigned the value of 100%.

**Figure A2:**
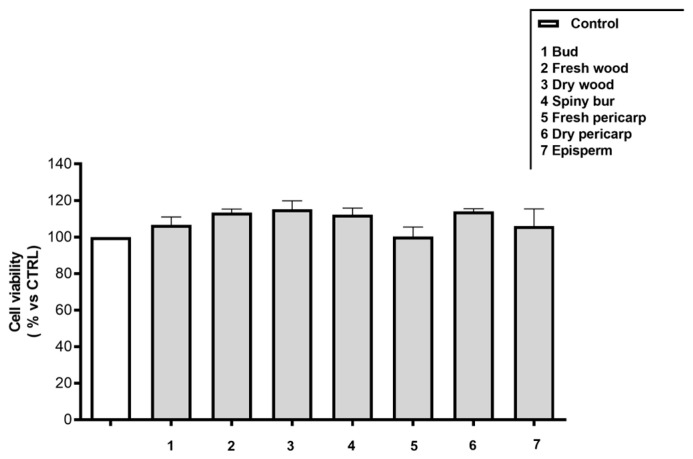
NRU assay performed on *C. sativa* by-product extracts (50 µg/mL) in CaCo-2. Data (*n* = 3) are expressed as mean (%) ± SEM relative to control, which was arbitrarily assigned the value of 100%.

Before assessing the anti-inflammatory activity in an intestinal cell model, all extracts were tested for their possible cytotoxic effects at 100 µg/mL. None of the extracts showed any cytotoxicity (Figure A1 and Figure A2). Therefore, the possible anti-inflammatory activity of *Castanea sativa* Mill. by-products on the release of several NF-κB inflammatory mediators following IL-1β-IFNγ-induced inflammation was explored.

**Figure 1:**
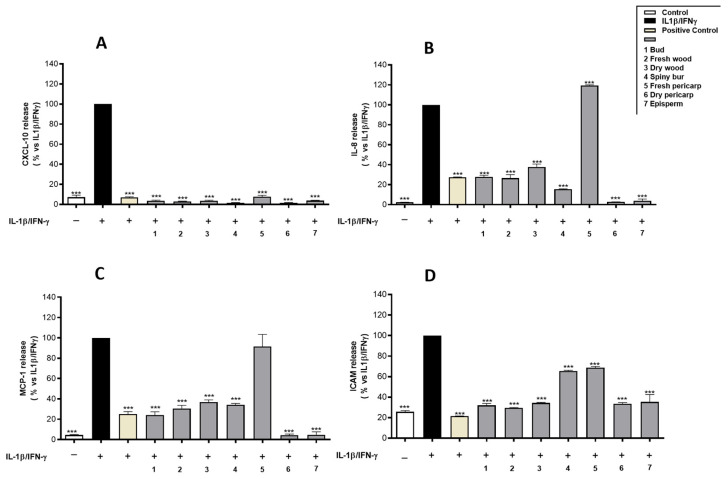
Effect of hydroalcoholic extracts from *C. sativa* by-products (50 µg/mL) on CXCL-10 (**A**), IL-8 (**B**), MCP-1 (**C**), ICAM-1 (**D**) release, measured by ELISA assay. Epigallocatechin-3-O-gallate (20 μM) was used as a reference inhibitor (positive control). Data (*n* = 3) are expressed as mean (%) ± SEM relative to IL-1β-IFN-γ, which was arbitrarily assigned the value of 100%. *** *p* < 0.001 vs. IL-1β-IFN-γ.

All the extracts were tested at 50 µg/mL for their inhibitory effects on the secretion of CXCL-10, IL-8, MCP-1, and ICAM-1, which are key mediators in the molecular pathways of intestinal inflammation [36,37]. The results, shown in Figure 1, indicate a statistically significant inhibition of the release of inflammatory mediators exerted by all the tested extracts, except for fresh pericarp, which did not inhibit IL-8 and MCP-1 secretion.

To elucidate the activity of the extracts on intestinal inflammation, the NF-κB- and HIF-driven transcription pathways were evaluated. NF-κB is a transcription factor involved in the regulation of the inflammatory response, controlling the transcription of proinflammatory cytokines, whose increased expression is linked to the pathogenesis of inflammatory bowel disease (IBD) [38].

**Figure 2:**
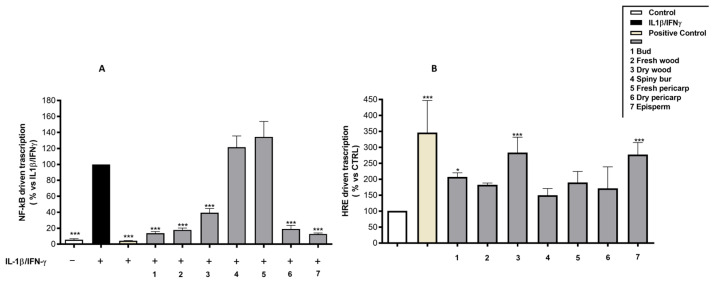
Effect of hydroalcoholic extracts from *C. sativa* by-products (50 µg/mL) on NF-κB- (**A**) and HRE (**B**)-driven transcriptions, measured by luciferase assay and reporter plasmids. DMOG (250 μM) was used as a reference compound for HIF induction (positive control) (**A**). Apigenin (20 μM) was used as reference inhibitor of the NF-κB activity (positive control). Data (*n* = 3) are expressed as mean (%) ± SEM relative to IL-1β -IFN-γ (**A**) or to control (**B**), which was arbitrarily assigned the value of 100%. * *p* < 0.05, *** *p* < 0.001 vs. IL-1β -IFN-γ (**A**) or Control (**B**).

Gut lumen is mostly a reducing and anaerobic environment, due to the activity of commensal bacteria, which are kept separated from the systemic circulation by gut barrier. HIF, a family of hypoxia-inducible factors, regulates cellular response to oxygen levels and has recently emerged as a new therapeutic target in IBD. HIF mitigates oxidative stress and tissue hypoxia by activating several cellular target genes, thereby helping to alleviate intestinal inflammation and to reinforce intestinal barrier integrity [39]. Thus, considering the involvement of HIF in intestinal diseases, it appeared interesting to test the effects of the extracts, at 50 µg/mL, on this parameter. The results showed that the hydroalcoholic extracts from buds and episperm exploited the highest inhibition on the NF-κB pathway among all the tested extracts, about −86.25% and −87.23% (vs. IL-1β-IFN-γ), respectively (Figure 2A). Spiny bur and fresh pericarp extracts, nevertheless, did not show any significant inhibition.

Previous studies, including those by our group, demonstrated the ability of chestnut by-products, such as leaves, pericarp, shells, and burs to modulate the pro-inflammatory transcriptional factor NF-κB. Cerulli et al. reported that a methanolic extract of *C. sativa* shells impaired NF-κB signaling following LPS stimulation in THP-1 monocytes [28]. Our group previously observed that the hydroalcoholic extracts of *C. sativa* pericarp and episperm inhibited the TNFα-induced NF-κB-driven transcription in AGS gastric epithelial cells in a concentration-dependent fashion [12]. However, to the best of our knowledge, the inhibition of this pathway in intestinal cells by chestnut products has not been investigated until now.

Here, a statistically significant induction of HIF-driven transcription was observed among episperm and dry wood extracts, suggesting their potential role in promoting intestinal barrier integrity (Figure 2B).

The inhibitory concentration (IC_50_) was therefore determined for the most promising parameters (i.e., CXCL-10, MCP-1, and IL-8 release vs. stimulus; NF-κB-driven transcription vs. stimulus) for each by-product (Table 3). Among the extracts, chestnut dry and fresh wood showed the highest inhibitory effect on CXCL-10, with IC_50_ values of 1.28 μg/mL (CI 0.78 to 2.09) and 1.43 μg/mL (CI 0.99 to 2.06), respectively. The inhibitory activity on NF-κB-driven transcription and MCP-1 secretion was more pronounced in dry pericarp and episperm extracts. The estimated IC_50_ values are 3.15 μg/mL (CI 2.07 to 4.77) and 6.42 μg/mL (CI 4.50 to 9.15) for MCP-1 secretion and 2.36 μg/mL (CI 1.12 to 5.02) and 2.27 μg/mL (CI 1.65 to 3.10) for NF-κB-driven transcription, respectively, for dry pericarp and episperm.

### 2.3. Identification of Promising Chestnut By-Products as Anti-Inflammatory Agents in the Gut

**Figure 3:**
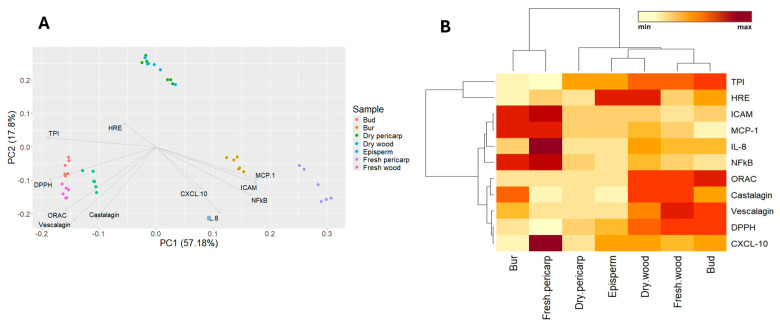
Classification of chestnut by-products for inflammation (MCP-1, ICAM-1, CXCL-10, IL-8 release vs. stimulus; NF-κB-driven transcription vs. stimulus), antioxidant activity (ORAC and DPPH), total polyphenol index, ellagitannin content (vescalagin and castalagin), HRE-driven transcription (induction % vs. CTRL), based on PCA (**A**) and cluster analysis (**B**).

The different behavior among extracts was analyzed by Principal Component Analysis (PCA) (Figure 3A), including all the variables related to inflammation, scavenging capacity, and chemical composition, with the purpose of identifying the most promising source of active principles. Two main components were identified, representing about 75% of the total variance. The first component (PC1) explained 57.18% of the variance and was positively influenced primarily by inflammatory variables (MCP-1 and ICAM-1 release vs. stimulus; NF-κB-driven transcription vs. stimulus) and negatively by antioxidant variables (ORAC and DPPH) and chemical composition (TPI, vescalagin). The second component explained 17.8% of the variance and was negatively influenced by scavenging capacity variables (ORAC and DPPH assays), chemical composition (ellagitannin content), inflammatory variables (MCP-1, ICAM-1, CXCL-10, IL-8 release vs. stimulus; NF-κB-driven transcription vs. stimulus) and positively influenced by HRE-driven transcription (induction % vs. CTRL) and TPI. According to these two main components, three main groups were identified: group 1, with spiny burs and fresh pericarp; group 2 included woody extracts, such as buds, fresh and dry wood; and group 3, which contained episperm and dry pericarp.

Chestnut by-product hydroalcoholic extracts were clustered according to TPI, antioxidant activity (DPPH, ORAC), ellagitannin content (vescalagin, castalagin), inhibition of inflammatory markers’ release (CXCL-10, IL-8, MCP-1, ICAM-1) vs. stimulus, inhibition of NF-κB-driven transcription vs. stimulus, and induction of HRE-driven transcription vs. CTRL. Using Euclidean distance, four groups were identified: group 1 contained burs and fresh pericarp; group 2 consisted of dry pericarp; group 3 included episperm and dry wood; group 4 comprised fresh wood and quiescent buds. 

Extracts belonging to group 1 showed high levels of inflammatory markers (MCP-1, ICAM-1, IL-8) and NF-κB, but low levels of DPPH, ORAC, and TPI. Groups 2, 3, and 4 exhibited low levels of inflammatory markers (MCP-1, ICAM-1, IL-8), with group 3 showing higher levels of HRE, while group 4 had high levels of ORAC, DPPH, TPI, vescalagin, and castalagin (Figure 3B).

The first main component was primarily associated with the secretion of pro-inflammatory mediators, whereas the second component was related to scavenging capacity and chemical composition. The best performance was observed in extracts belonging to groups 3 and 4. Specifically, quiescent bud extract from group 4 showed the lowest release of inflammatory mediators (CXCL-10, MCP-1, ICAM-1, IL-8), the highest scavenging capacity (ORAC and DPPH), and highest chemical composition (TPI, ellagitannins); moreover, it also inhibited the NF-κB-driven transcription. Dry wood extract form group 3 showed similar patterns to bud extract regarding chemical composition, scavenging and anti-inflammatory activities, and the greatest induction of HRE-driven transcription.

### 2.4. Stability of Promising Chestnut By-Products

According to cluster analysis, quiescent buds and dry wood were identified as the most promising extracts for potentially counteracting inflammatory intestinal diseases among all chestnut by-products. To evaluate the stability of these promising by-products, a gastrointestinal digestion was carried out. The parameters evaluated on digested extracts included the following: CXCL-10 secretion for anti-inflammatory activity, DPPH assay for scavenging capacity, and total polyphenol index.

**Figure 4:**
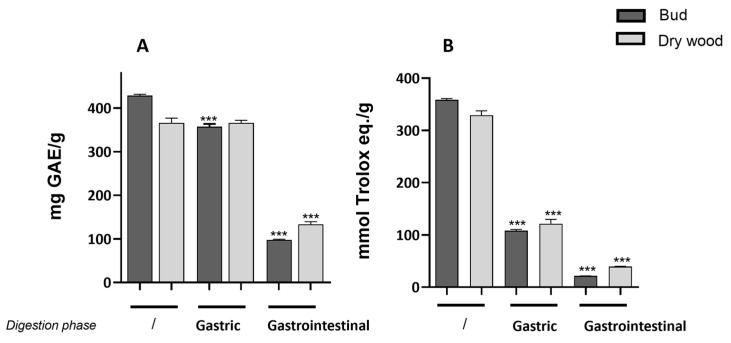
Effect of gastric or gastrointestinal digestion on total polyphenol index (mg gallic acid equivalent/g ± SE) and antioxidant activity (mmol Trolox equivalent/g ± SE) measured by DPPH assay on *C. sativa* bud and dry wood extract. Data (*n* = 3) are expressed as mean ± SEM. *** *p* < 0.001 vs. undigested extracts.

Gastric phase showed a statistically significant decrease in total polyphenol index compared to the undigested extracts only in buds. At the intestinal level, a statistically significant reduction in polyphenol content was noted in both bud and dry wood extracts (Figure 4A).

Scavenging capacity in chestnut bud and dry wood following simulated gastrointestinal digestion is reported in Figure 4B. Both extracts exhibited a similar and statistically significant reduction in scavenging capacity compared to the undigested extracts. 

**Figure 5:**
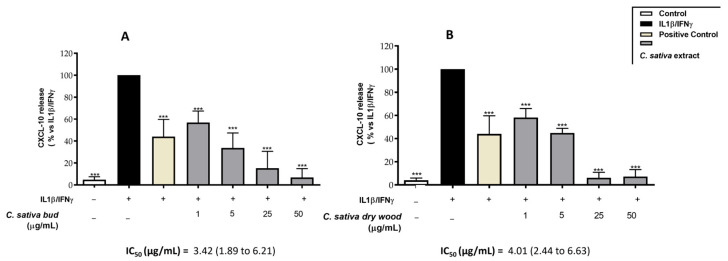
Effect of *C. sativa* bud (**A**) and dry wood (**B**) extracts after in vitro gastrointestinal digestion on CXCL-10 release, measured by ELISA assay. Epigallocatechin-3-O-gallate (20 μM) was used as reference inhibitor (positive control, in yellow). Data (*n* = 3) are expressed as mean (%) ± SEM related to IL-1β-IFN-γ, which was arbitrarily assigned the value of 100%. *** *p* < 0.001 vs. IL-1β-IFN-γ.

CXCL-10 secretion by CaCo-2 cells after IL-1β-IFN-γ-induced inflammation was significantly reduced in a concentration-dependent way by both gastrointestinally digested extracts (Figure 5A,B). The IC_50_ of the extracts was about 3.42 µg/mL (1.89–6.1) (CI 95%) and 4.01 (2.44–6.63) (CI 95%) for bud and dry wood, respectively. These results are in line with the IC_50_ values observed in bud and dry wood extracts before digestion, equal to 2.13 µg/mL (1.68–2.70) (CI 95%) and 1.28 µg/mL (0.78–2.09) (CI 95%) (Table 4).

Despite the significant reduction in the TPI of *C. sativa* by-products following gastric and intestinal digestion, with a reduction of up to 50% in the intestinal phase, the bioactivity of these extracts on both scavenging and anti-inflammatory parameters remained promising.

## 3. Materials and Methods

### 3.1. Materials

Folin–Ciocalteu’s reagent, 1,1-diphenyl-2-picryl-hydrazyl free radical (DPPH), salts, the reagents 3-(4,5-dimethylthiazol-2-yl)-2,5-diphenyltetrazolium bromide (MTT), and 3-amino-7-dimethylamino-2-methyl-phenazine hydrochloride (Neutral Red), gallic acid, DMOG (dimethyloxalylglycine), fluorescein, Trolox(R) were purchased from Merck Life Science (Merck Life Science, Milan, Italy). Lipofectamine^®^ 3000 reagent was acquired from Invitrogen (Thermo Fisher Scientific, Monza, Italy). Britelite^TM^ Plus reagent was obtained from Revvity (Revvity, Milan, Italy). The ellagitannins castalagin and vescalagin (certified purity, >95%), apigenin, and epigallocatechin-gallate (EGCG) were procured from Phytolab (Phytolab GmbH & Co. KG, Vestenbergsgreuth, Germany). DMEM medium, trypsin, streptomycin, non-essential amino acids, sodium pyruvate, and L-glutamine were purchased from Gibco^TM^ (Thermo Fisher Scientific, Monza, Italy), while Fetal Bovine Serum (FBS) and disposable materials for cell culture were from Euroclone (Primo^®^, Euroclone S.p.a., Pero, Italy). Well plates for experiments were from Corning (Falcon^®^; Corning Life Sciences, Amsterdam, The Netherlands). All reagents used for the biological assays were of HPLC grade. Human IL-1β and human IFN-γ were sourced from Peprotech (Peprotech Inc., London, UK). All chromatographic solvents were of HPLC grade or LC–MS grade for MS experiments. Acetonitrile, methanol, ethanol, and formic acid were purchased from Carlo Erba (Carlo Erba, Milan, Italy).

### 3.2. Sample Harvesting and Extraction

All chestnut by-products were collected from the farmer consortium in the regional area of Campo dei Fiori (VA), Italy. Quiescent chestnut buds and wood obtained from pruning were collected in March 2022. Spiny burs and the external parts of the fruit (episperm and pericarp) were collected in October 2022.

Buds, fresh and dry wood, spiny burs, fresh and dry (30 min at 140 °C) pericarp and episperm were manually ground under liquid nitrogen; 2 g was suspended in 20 mL of a 50:50 ethanol/water solution, vortexed for 1 min, sonicated for 30 min, centrifuged at 2000 rpm for 5 min, and the supernatant was recovered. This procedure was repeated twice; the recovered supernatants were filtered, dried, and dissolved in DMSO for the biological assays. The extracts were diluted in MeOH for LC-MS/MS analysis. The extraction yields for chestnut by-products were 17.5% (fresh wood), 18.2% (dry wood), 14.4% (bud), 21.6% (bur), 19.8% (dry pericarp), 16.3% (fresh pericarp) and 17.8% (episperm).

### 3.3. Extract Characterization

#### 3.3.1. Total Polyphenol Index (TPI)

Samples from plant extracts were analyzed by Folin–Ciocalteu assay to obtain the TPI. Firstly, they were properly diluted in water, to a final volume of 800 μL; then 50 μL of 2 N Folin–Ciocalteu reagent, and 150 μL of 20% (*w*/*v*) Na_2_CO_3_ were added. Samples were incubated for 30 min at 37 °C, and then transferred into plastic cuvettes to measure their absorbance at a wavelength of 765 nm by using a Jasco V630 Spectrophotometer (Jasco Europe s.r.l., Lecco, Italy). Gallic acid was used as reference polyphenol to build a calibration curve.

#### 3.3.2. Quantification of Ellagitannins

LC-MS/MS quantification of individual molecules was carried out according to our previous work [11]. Briefly, the ellagitannins castalagin and vescalagin were quantified through an Exion LCTM AC System (AB Sciex, Milano, Italia) and a Triple Quad^TM^ 3500 system (AB Sciex, Milano, Italia) in ESI negative ionization. The natural compounds were separated on a Phenomenex Synergi Hydro-RP (80 Å, 4 μm, 150 × 4.6 mm) column. The mobile phase was composed of 0.1% formic acid in water (A) and methanol (B), with a rate flow set on 0.800 mL/min. Calibration curves were made by serial dilutions of vescalagin and castalagin in MeOH, ranging from 0 to 2.5 ng/µL. Their equations were y = 19.459x^2^ + 4360.1x − 3358, R^2^ = 0.998 for vescalagin, and y = 164.48x^2^ + 27,601x − 18,420, R^2^ = 0.999 for castalagin, respectively.

#### 3.3.3. Polyphenolic Fingerprint 

The polyphenolic profile of chestnut by-product extracts was acquired by an analytical approach based on HPLC (Surveyor MS PUMP PLUS system, Thermo Fisher Scientific, Monza, Italy) coupled to mass spectrometry (LTQ ion trap, Thermo Fisher Scientific, Monza, Italy) using an ESI (ElectroSpray Ionization) source in negative mode. The analytes were separated on a Phenomenex Synergi Hydro-RP (80 Å, 4 μm, 150 × 4.6 mm) column with a mobile phase composed of 0.1% water and formic acid (phase A) and acetonitrile (phase B) at a rate flow of 0.600 mL/min. The following elution gradient was applied: minute 0 95% A–5% B, minute 1 95% A–5% B, minute 10 100% A–0% B, minute 15 100% A–0% B, minute 15.10 95% A–5% B, minute 25 95% A–5% B. The extracts were injected at an amount of 0.1 mg/mL, and the peaks obtained were analyzed using the Xcalibur™ Software 4.3—Thermo Fisher Scientific by selecting only peaks with an intensity greater than 10^2^. The compounds contained in the plant matrix were putatively identified by comparing the mass spectra obtained with the *m*/*z* ratios and related fragmentations to the ones found in literature [10,33].

### 3.4. In Vitro Evaluation of the Anti-Inflammatory Activity

#### 3.4.1. Cell Culture and Treatment

Intestinal epithelial cells from human colorectal cancer (CaCo-2, clone HB237), obtained by the American Type Culture Collection (ATCC, Manassas, VA, USA), were cultured in DMEM medium (supplemented with 100 mg streptomycin, 100 units penicillin per mL, 1% non-essential amino acids, 1 mM sodium pyruvate, 4 mM l-glutamine, and 10% Fetal Bovine Serum). For the cultivation and the experimental procedures, the cells were detached from 75 cm^2^ flask using Trypsin-EDTA 0.25%, counted, and transferred into new flasks or plates. The cells were always incubated under a humidified atmosphere with 5% CO_2_ at 37 °C, by avoiding the confluence. 

For the treatments, CaCo-2 cells were cultured for 48 h in 24-well plates (3 × 10^4^/well). On the day of treatment, the cells were stimulated with a pro-inflammatory combination of IL-1β (10 ng/mL) and IFN-γ (10 ng/mL), following well-established procedures [36]. The read-out markers were evaluated after 6 or 24 h of stimulation and simultaneously treated with chestnut by-product extracts, according to the inflammatory parameter assessed.

#### 3.4.2. Cell Viability

CaCo-2 cells were observed before and after each treatment by light microscope inspection to exclude the alteration in cell morphology. Cell viability was measured using the 3,4,5-dimethylthiazol-2-yl-2-5-diphenylte-trazolium bromide (MTT), as previously described [11]. Briefly, MTT solution (Phosphate Buffer Solution, PBS 1X, 200 µg/mL) was prepared and added to cells after 24 h of incubation with chestnut by-product extracts. The samples were then lysed with an isopropanol/DMSO (90:10 *v*/*v*) solution after 30 min of MTT metabolization, to dissolve the purple formazan salt, which had been formed in viable and metabolically active cells. The absorbance was read at a wavelength of 550 nm and directly correlated with cell viability. 

The Neutral Red Uptake (NRU) was selected as a further viability assay, according to Repetto et al. [40]. Briefly, 3-amino-7-dimethylamino-2-methyl-phenazine hydrochloride (Neutral Red) solution (40 µg/mL) was added to the cells after removing culture medium. The cells were incubated for 2 h at 37 °C; then, the medium was discarded and the cells washed with PBS 1X. To obtain measurable samples, a destaining solution (EtOH 50% *v*/*v* + 1% *v*/*v* glacial acetic acid) was added to the cells, and its absorbance was measured at 535 nm by using a multiplate spectrophotometer (Envision, Perkin Elmer, Milan, Italy).

#### 3.4.3. Measurement of Inflammatory Markers

Human IL-8 (CXCL8), Human IP-10 (CXCL-10), Human MCP-1 (CCL2), and Human ICAM-1 ELISA development ABTS kits were purchased from PeproTech (PeproTech Inc., London, UK) and used to measure the release of inflammatory mediators by CaCo-2 cells after 24 h of treatment. ELISA assays were conducted according to the manufacturer’s instructions, as previously reported [11]. The day before, Corning 96-well EIA/RIA plates (Merck Life Science, Milan, Italy) were coated with the capture antibodies. Then, the levels of inflammatory proteins were measured in the culture media by reading the absorbance (405 nm) obtained from the colorimetric reaction between 2,2′-Azino-bis(3-ethylbenzothiazoline-6-sulfonic acid) (ABTS) substrate (Merck Life Science, Milan, Italy) and horseradish peroxidase enzyme (VICTOR X3; PerkinElmer, Milan, Italy). The obtained results (mean ± SEM of at least three experiments) were expressed as a percentage relative to the stimulated control, which was arbitrarily assigned the value of 100%. EGCG (20 μM) was chosen as reference anti-inflammatory polyphenol, according to previously published studies concerning its bioactivity against intestinal inflammation [41].

#### 3.4.4. Measurement of the NF-κB-Driven Transcription 

NF-κB-driven transcription was evaluated as previously reported [36]. Briefly, CaCo-2 cells were transiently transfected with a reporter plasmid (NF-κB Luc) responsive to NF-κB (250 ng per well). The plasmid contains the luciferase gene under the control of the E-selectin promoter, which is characterized by three κB responsive elements. To carry out the transfection, Lipofectamine® 3000 Reagent was used. The plasmid was a gift from Dr. N. Marx (Department of Internal Medicine-Cardiology, University of Ulm; Ulm, Germany). The day after, the cells were treated with IL-1β (10 ng/mL) and IFN-γ (10 ng/mL), in addition to chestnut by-product extracts (50 μg/mL) for 6 h. Apigenin (20 μM) was used as a reference inhibitor. Britelite^TM^ Plus reagent was used to assess the amount of luciferase produced into the cells, according to the manufacturer’s instructions. A VICTOR X3 Multilabel Plate Reader (Perkin Elmer, Milan, Italy) was used to measure the consequent development of luminescence. The results (mean ± SEM of at least three experiments) were expressed as percentage relative to the stimulated control, which was arbitrarily assigned the value of 100%.

#### 3.4.5. Measurement of the HRE-Driven Transcription

CaCo-2 cells were transiently transfected with a reporter plasmid responsive to HIF-1α (250 ng per well), containing HRE-Luc, a luciferase reporter construct containing three hypoxia-responsive elements (24-mers) from the Pgk-1 gene Lipofectamine^®^ 3000 transfection reagent, which was used for transfection assays. The plasmid was a gift from Navdeep Chandel to Addgene repository (Addgene plasmid # 26731; http://n2t.net/addgene:26731, accessed on 17 January 2024; RRID: Addgene_26731). The following day, the cells were incubated with chestnut by-product extracts (50 μg/mL) for 6 h. Dimethyloxalylglycine (DMOG) (250 μM) was used as a reference compound for HIF induction. As previously described, Britelite^TM^ Plus reagent was used to assess the amount of luciferase produced into the cells; then, VICTOR X3 Multilabel Plate Reader (Perkin Elmer, Milan, Italy) was used to measure the luminescence. The results (mean ± SEM of at least three experiments) were expressed as a percentage relative to the control, which was arbitrarily assigned the value of 100%.

#### 3.4.6. Gastrointestinal Digestion

The in vitro gastrointestinal digestion was simulated as described by Sangiovanni et al. [42] with minor modifications; briefly, 0.6 g of chestnut by-products extracts was suspended in 6 mL of saliva and stirred at 37 °C. After 5 min, 12 mL of gastric juice was added to the suspension, and the solutions were stirred for 2 h at 37 °C. Finally, to simulate the intestinal phase, 12 mL of duodenal juice and 6 mL of bile were added, and the solutions were stirred for 2 h at 37 °C. At the end of the procedure, the solutions were freeze-dried and stored at −20 °C for cell treatments. A blank sample containing only the digestive enzymes was provided to consider the possible interference in the biological assays.

### 3.5. Scavenging Capacity

#### 3.5.1. ORAC Test

The oxygen radical absorbance capacity (ORAC) assay was carried out according to Fracassetti et al. [43]. In brief, an aliquot from stock solutions of chestnut by-product extracts was distributed into a black 96-well plate and diluted to a volume of 20 μL. Then, 120 μL of fluorescein solution (final concentration equal to 70 nM), previously prepared with phosphate buffer (pH 7.4, 75 mM), was added to each well. Peroxyl radicals were generated by adding 60 μL of AAPH 40 mM (Merck Life Science, Milan, Italy). The plate was placed in a multiplate reader (Victor X3, PerkinElmer, Milan, Italy) to measure the fluorescence (excitation/emission: 484/528 nm) after shaking every 2 min for 60 min at 37 °C. Trolox (0–120 μM) was used as a reference inhibitor. The area under the curve (AUC) of each extract was calculated and the results were expressed as mmol Trolox equivalent per g of powder.

#### 3.5.2. DPPH Test

DPPH test was performed according to Fracassetti D., et al. [43]. Briefly, dried plant extracts were dissolved in 70% methanol, centrifugated, and serially diluted. Fresh DPPH solution was diluted with methanol to obtain 1.00 ± 0.03 absorbance units at 515 nm. Then, samples were prepared in a 96-well microplate: 245 μL of DPPH solution was placed in each well and 5 μL of the sample was added. After 50 min, each sample was measured by a microplate reader (Envision, Perkin Elmer, Waltham, MA, USA). A calibration curve was made by adding an increasing concentration of Trolox ranging from 0 to 2.5 mmol. Each concentration was assayed in triplicate, as well as each sample. The results were expressed as mmol Trolox equivalents per g of powder.

### 3.6. Statistical Analysis

All data were expressed as the mean ± SEM of at least three independent experiments. ELISA assays were analyzed by unpaired one-way analysis of variance (ANOVA), followed by the Bonferroni post hoc test. Chemical characterization data and antioxidant assays were analyzed by analysis of variance (ANOVA), followed by the Duncan post hoc test. The correlation between variables was assessed by Pearson’s linear correlation. Statistical analysis was performed using SPSS statistical environment (IBM SPSS Statistics 29) and GraphPad Prism 9.0 software (GraphPad Software Inc., San Diego, CA, USA). Values of *p* < 0.05 were considered statistically significant. For ANOVA models, the levels of significance were considered as follows: *p* < 0.05 (*); *p* < 0.01 (**); *p* < 0.001 (***). Cluster analyses and a heatmap were performed using the Euclidean distance and a complete linkage method. Principal component analysis (PCA) was carried out using the ggplot2, ggfortify, and ggrepel R packages 4.1.2.

## 4. Conclusions

This study offers valuable insights into the potential application of chestnut by-products to counteract intestinal inflammation. The presence of bioactive compounds, particularly polyphenols, and the observed scavenging capacity and anti-inflammatory activities suggest a promising application of these by-products or related compounds as novel therapeutic agents for intestinal inflammatory diseases. Furthermore, this research significantly contributes to the valorization of plant waste derived from both agronomic and industrial practices. In vitro gastrointestinal digestion of the most promising extracts confirmed the partial stability of these by-products, which maintain their scavenging and anti-inflammatory activities as well as a high polyphenol content. Bud and dry woods extracts showed the best biological activities, with all the IC_50_ values for the tested mediators of inflammation below 10 μg/mL. Future research will aim to explore the mechanisms underlying the observed biological activity and to identify the specific compounds or molecules responsible. Subsequent clinical trials and preclinical models will play a pivotal role in confirming and validating these promising biological activities.

## Figures and Tables

**Table 1:** Summary of TPI (mg gallic acid equivalent/g ± SEM), scavenging capacity assays (mmol Trolox equivalent/g ± SEM), and ellagitannin content (µg/g ± SEM) of *C. sativa* by-product extracts.

	**TPI ^1^**	**ORAC Assay**	**DPPH Assay**	**Vescalagin**	**Castalagin**
	(mg GAE ^2^/g ± SEM ^3^)	(mmol Trolox Equivalent/g ± SEM)	(mmol Trolox Equivalent/g ± SEM)	(µg/g ± SEM)	(µg/g ± SEM)
Bud	428.4 ± 3.3 a	51 ± 1.8 c	7.2 ± 0 f	19.2 ± 0.1 b	20.5 ± 0.0 d
Fresh wood	391.2 ± 1.3 b	46 ± 1.7 b	7.3 ± 0.1 f	21.4 ± 0.2 a	41.3 ± 0.2 a
Dry wood	365.6 ± 11.4 c	47 ± 1.0 b	6.6 ± 0.2 e	12.7 ± 0.0 c	39.4 ± 0.1 b
Dry pericarp	287.2 ± 8.4 e	17.8 ± 0.0 a	2.77 ± 0.07 c	0.4 ± 0.0 e	6.9 ± 0.1 e
Fresh pericarp	54.9 ± 7.3 g	16.4 ± 10.1 a	0.17 ± 0.03 a	0.29 ± 0.0 e	2.11 ± 0.0 g
Episperm	314.3 ± 12.1 d	17.3 ± 0.1 a	3.11 ± 0.14 d	0.34 ± 0.0 e	3.55 ± 0.0 f
Spiny bur	117.5 ± 7.0 f	17.1 ± 0.1 a	1.39 ± 0.14 b	7.4 ± 0.7 d	32.4 ± 0.5 c

^1^ Total polyphenol index evaluated by Folin–Ciocalteu method; ^2^ GAE: Gallic acid equivalent; ^3^ SEM: Standard error of three independent experiments. Data are expressed as mean values ± SEM. Different letters denote significant differences (*p* < 0.05).

**Table 2:** Metabolites putatively identified in the hydroalcoholic extract of *C. sativa* buds (B), spiny burs (SB), wood (W), pericarp (P), episperm (E).

N	RT	(M-H)	Product Ion (*m*/*z*)	Putative Identified Molecule	B	SB	W	P	E
1	3.18	191	129, 146, 159 173	quinic acid	x		x		
2	5.31	337	293	hexahydroxydiphenic acid	x		x		
3	5.49	609	305, 423, 441, 483, 591	condensed tannin GC-GC-B type				x	
4	5.60	933	411, 631, 915	castalagin/vescalagin				x	
5	5.99	317	136, 155	phenol glucoside (crenatin)			x		
6	5.63	609	305, 423, 441, 483, 591	condensed tannin GC-GC-B type					x
7	6.12	933	658, 897, 899	castalagin/vescalagin	x	x	x		
8	6.13	935	481, 782, 863, 915, 916, 917	stachyurin		x			
9	6.63	613	299, 300, 493, 494	castacrenin B		x			
10	6.95	483	169, 211, 241. 270, 312, 331, 415, 465	digalloyl glucose isomer			x		
11	6.98	933	348, 451, 631	castalagin, vescalagin					x
12	7.18	289	179, 205, 245	catechin				x	
13	7.22	635	313, 465, 483, 603, 604	trigalloyl glucose isomer			x		
14	7.23	637	305, 467, 469, 593	chesnatin			x		
15	7.83	937	467, 469, 637	chestanin			x		
16	7.96	469	168, 425	cretanin			x		
17	8.34	621	313, 317, 451, 469, 577	galloyl cretanin			x		
18	9.13	433	300, 301, 302, 369	ellagic acid pentoside	x	x	x		
19	9.61	301	184, 201, 228, 257, 283	ellagic acid		x			
20	9.67	447	315, 316	astragalin			x		
21	9.81	343	297, 312, 328, 329	trimethylellagic acid		x			
22	9.85	461	315, 328, 446	dimethylellagic acid pentoside		x			
23	10.82	329	314	dimethylellagic acid	x	x			
24	11.53	343	298, 313, 328, 329	trimethylellagic acid	x			x	
25	15.18	461	222, 445, 446	dimethylellagic acid pentoside	x				

The presence of natural compounds specified above was marked by “x”.

**Table 3:** IC_50_ values of *C. sativa* by-product extracts on the CXCL-10, IL-8, and MCP-1 release, and NF-κB-driven transcription induced by IL-1β/IFN-γ in CaCo-2 cells.

	CXCL-10		IL-8		MCP-1		NF-κB	
	IC_50_ (μg/mL)	CI (95%)	IC_50_ (μg/mL)	CI (95%)	IC_50_ (μg/mL)	CI (95%)	IC_50_ (μg/mL)	CI (95%)
Bud	2.13	1.68–2.70	10.10	7.26–14.05	8.01	5.55–11.55	14.65	13.71–15.67
Bur	4.50	3.58–5.66	3.67	2.68–5.01	25.37	18.17–35.43	n.d.	
Fresh Wood	1.43	0.99–2.06	7.58	5.12–11.21	13.08	8.63–19.82	17.49	12.36–24.74
Dry Wood	1.28	0.78–2.09	21.26	14.65–30.86	23.81	18.08–31.35	31.96	27.93–36.58
Fresh Pericarp	7.34	6.03–8.94	n.d.		n.d.		n.d.	
Dry Pericarp	1.82	1.14–2.91	8.01	6.69–9.59	3.15	2.07–4.77	2.36	1.12–5.02
Episperm	1.64	0.95–2.82	3.91	2.83–6.42	6.42	4.50–9.15	2.27	1.65–3.10

Data are expressed as mean values with CI (95%). IC_50_ (μg/mL), 50% inhibitory concentration; CI, confidence interval; n.d. not detectable.

**Table 4:** IC_50_ values of *C. sativa* by-product extracts before and after gastrointestinal digestion on the CXCL-10 release induced by IL-1β/IFN-γ.

	Before Digestion		After Digestion	
CXCL-10	IC_50_ (μg/mL)	CI (95%)	IC_50_ (μg/mL)	CI (95%)
Bud	2.13	1.68–2.70	3.42	1.89–6.21
Dry Wood	1.28	0.78–2.09	4.01	2.44–6.63

Data are expressed as mean values with CI (95%). IC_50_ (μg/mL), 50% inhibitory concentration; CI, confidence interval.

## Data Availability

Data are available on request from the corresponding authors stefano.piazza@unimi.it (S.P.) and mario.dellagli@unimi.it (M.D.A.).
